# Solar irradiation on the rear surface of bifacial solar modules: a modeling approach

**DOI:** 10.1038/s41598-020-70235-3

**Published:** 2020-08-06

**Authors:** Beyza Durusoy, Talat Ozden, Bulent G. Akinoglu

**Affiliations:** 1 Physics Department, Middle East Technical University (METU), 06800 Ankara, Turkey; 2 Department of Energy Systems Engineering, Gumushane University, 29000 Gumushane, Turkey; 3 The Center for Solar Energy Research and Applications (GÜNAM), METU, Ankara, Turkey; 4 Earth System Science Program, METU, Ankara, Turkey

**Keywords:** Renewable energy, Devices for energy harvesting

## Abstract

The transition in the energy sector has started with the growing population leading to the growing energy demands. The use of photovoltaic (PV) technologies has become a crucial way to meet energy demand. There are many ongoing studies for increasing the efficiency of commercial PV modules. One way to increase the energy yield of the PV modules is to use bifacial solar panels by capturing the rear side illumination as well. One of the challenges for estimating the bifacial module performances is to calculate the solar irradiation impinging on the rear side. Many models presented up to now require high computational power, and they are challenging to implement real-life conditions. In this paper, a simple physical modeling approach is presented to calculate the rear side solar irradiation incident on the bifacial modules. For the rear side irradiance estimation, the maximum difference between the measured and calculated rear side irradiance value is approximately 10 W/m^2^. The model does not require high computational skills since it is neither focused on the view factor nor ray tracing methodologies but instead uses solar geometry. The yield of the module is also modeled, calculated, and compared with the measurements.

## Introduction

The growing energy demand leads to the transition in the energy sector, and renewable energy has gained importance. Since solar energy is one of the most significant sustainable sources, photovoltaic technology dominates the renewable energy market. There are commercially available software programs such as PVSYST, PV*Sol, Helioscope, and PVWatts to assess the performance of the photovoltaic system^1^. However, modeling the field performance of a bifacial PV system is much complicated since estimating the rear side irradiation depends not only on the location and system design but also on installation conditions such as the tilt angle, the elevation of the module, and the albedo of the ground^2^. For this reason, it is necessary to know the effects of these parameters to predict the energy yield of the system.

The most crucial factor for a PV system to function at its maximum potential is the amount of solar radiation received. The total solar irradiation, namely, global solar irradiation, consists of beam, diffuse, and ground reflected irradiation^3^. Meteorological stations usually provide data for global solar irradiation on a horizontal surface. After determining the beam and diffuse components of global solar irradiation on a horizontal surface, tilted versions of these components can be deduced^4^. For this purpose, there are several models available to estimate the solar irradiation on a tilted PV module.

Compared to the monofacial PV modules, the energy yield of bifacial PV modules is up to 25% more than monofacial PV since bifacial PV modules can capture rear side irradiation as well. Although bifacial PV technology arises in the 1960s^5^, there is still no standard testing method yet. The reason is that there are lots of installation and location-dependent parameters that affect the rear side irradiation^6^. Up to now, there are few attempts to model the performance of bifacial PV modules based on two conventional approaches: View factor and ray-tracing methods. Although these methods are useful for specific site conditions, they both require high computational power^7,8^. Besides, most of the available models for bifacial PV modules ignore the contribution of beam radiation on the rear sides. However, when the angle of incidence of beam irradiation is greater than 90°, the Sun is behind the surface^9^, meaning that the rear side of the bifacial module receives beam radiation as well. The estimation of rear side irradiation is rather complicated due to the contribution of ground reflected radiation since that component is highly dependent on the location, surroundings, the reflection coefficient of the ground (albedo), and the elevation of the module. The presented model includes the beam radiation in the estimation of the rear side irradiation.

In this paper, we present a simple physical modeling approach to calculate the rear side irradiation incident on a single bifacial PV module. The energy yield of the bifacial PV module is calculated by using the presented model and by a modified yield calculation scheme. The model applies to any installation/site conditions, and the model does not require high computational power, unlike its predecessors.

## Data, methodology and model description

### Test site and module

The test site is on the rooftop of the Physics department at METU, Ankara (Central Anatolia). The climate of the test site is hot and semi-arid warm temperate^10^. The latitude of Ankara is 39.9°N, and the longitude is 32.8°E. The yearly total precipitation for the last 30 years is 388.1 mm. The average monthly temperature is about 12 °C. For July and August, the maximum average temperature rises to 30 °C and for January, the minimum average temperature drops to − 3 °C (State Meteorological Service of Turkey; https://www.mgm.gov.tr ).

**Figure 1:**
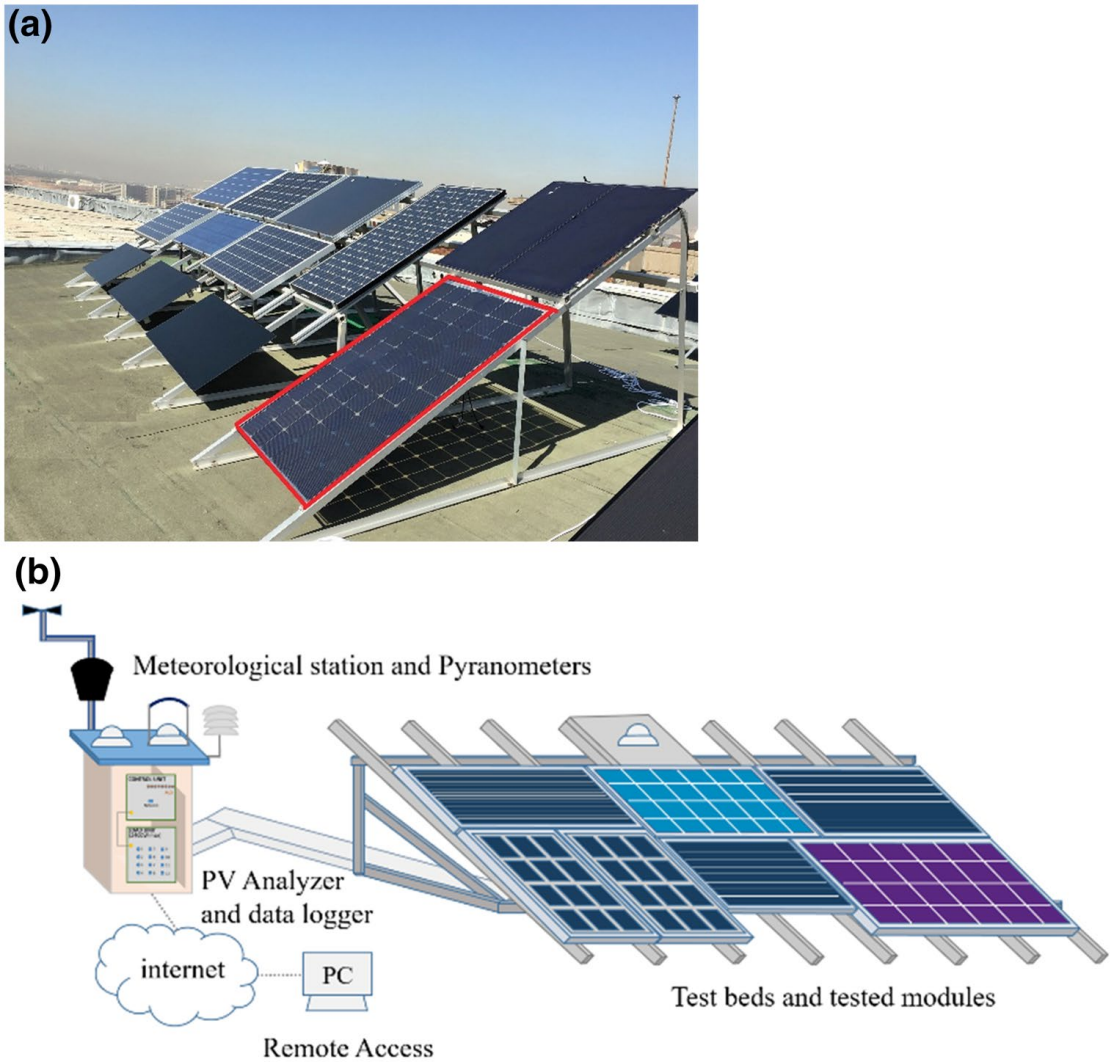
(**a**) Outdoor Test Facility in GUNAM, METU. A red square indicates the tested bifacial module. (**b**) Representation of all components in the test facility.

The test platform has 16 testbeds for monitoring the performance of PV modules. These testbeds are convenient for different types and frame structure of PV modules. The facility at the rooftop of the Physics Department in the METU campus is below in Fig. 1, and the bifacial module tested in this study is indicated by red lines. The test platform has a 32° tilt angle, and the tilt angle of the PV systems is the same as the testbeds.

In the Outdoor Test Platform, Daystar Multi Tracer 5 measures PV performance parameters and module temperatures by using the thermocouples, one of which is connected to the bifacial module of the present work. There are two pyranometers that we use them to measure horizontal and on-plane irradiation. We used these two pyranometers in the present work. We measure solar irradiation on a horizontal surface with a Davis weather station as well. The test device Daystar can measure each module's performance using both average and instantaneous parameters at a 10-min time interval. Table 1 shows the measured parameters. The data is easily accessible with the use of the FTP protocol.

**Table 1:** Summary of the parameters that the test device measures for the performance assessment of PV modules.

Measurement type	Parameters
Instantaneous	*V*_*oc*_ (V), *V*_*max*_ (V),* I*_*sc*_ (A),* I*_*max*_ (A),* P*_*max*_ (W),* FF* (%) *T*_*ambient*_ (°C), *T*_*module*_ (°C), *Irradiation*_*horizontal*_ (W/m^2^), *Irradiation*_*tilted*_ (W/m^2^)
Average	*V*_*max*_ (V),* I*_*max*_ (A),* P*_*max*_ (W),* T*_*ambient*_ (°C),* T*_*module*_ (°C) *Irradiation*_*horizontal*_ (W/m^2^),* Irradiation*_*tilted*_ (W/m^2^)

We have recorded all the meteorological data since 2015. The weather stations can measure rainfall (mm), pressure (millibar), ambient temperature (°C), relative humidity (%), horizontal total solar radiation (W/m^2^), UV dose (MEDs), wind speed (m/s) and direction (°). In this study, we have used a single bifacial PV module. Table 2 shows the characteristics of the module. The length and the width of the module are 1.61 m and 1.0 m, respectively. The lowest point of the module is 0.5 m above from the gray concrete ground with an albedo of 0.2.

**Table 2:** Summary of the properties of the bifacial PV module.

Module types	P_NOM_ (W)	P_MAX_ (W)	V_OC_ (V)	I_SC_ (A)	V_MPP_ (V)	I_MPP_ (A)	Tilt_angle_ (°)	Area (m^2^)
Bifacial	290.0	294.6	44.10	8.8	35.7	8.3	32	1.61

**Figure 2:**
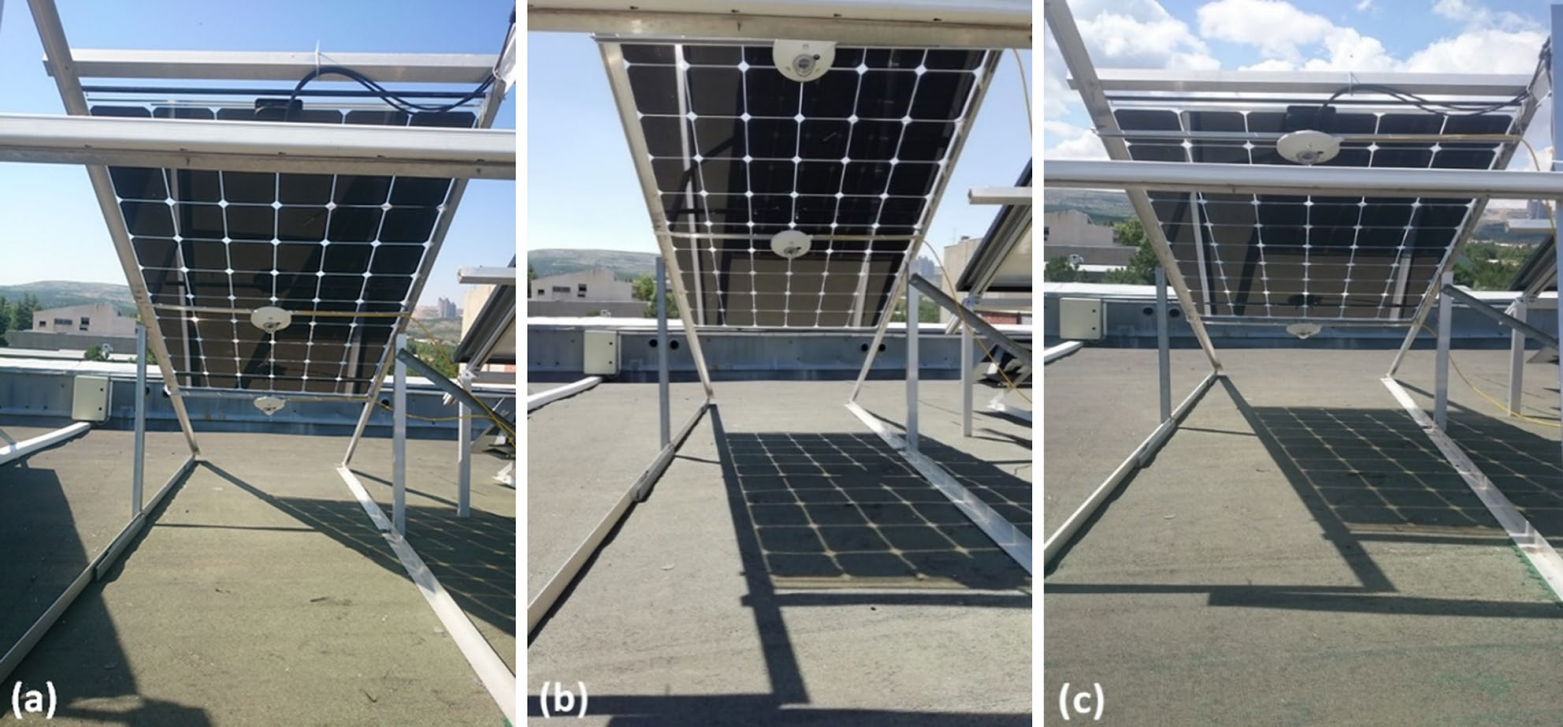
(**a**) The first configuration with one pyranometer is at the bottom-back (0.50 m), and the other is at the middle-back (0.84 m). (**b**) The second configuration with one pyranometer is at the top-back (1.16 m), and the other is at the middle-back (0.84 m). (**c**) The third configuration with one pyranometer is at the bottom-back (0.50 m), and the other is at the top-back (1.16 m).

We have constructed three different setups to observe the effect of elevation on the ground reflected radiation received by the rear side of the bifacial PV module. Placing two pyranometers at the rear side of the bifacial PV module at three different height levels, two levels at a time, we measured the rear side irradiance, respectively. Figure 2 shows three configurations of the pyranometers on the rear side of the bifacial PV module. The data includes three typical days for three configurations. The first configuration involves one pyranometer in the middle and the other at the bottom: 0.84 and 0.50 m above the ground. For the second configuration, we have placed the pyranometer at the bottom to the top 0.50 and 1.16 m above the ground. As a final step, for the third configuration, we removed the one in the middle and placed it to the bottom: 1.16 and 0.50 m above the ground.

### Introduction to the methodology

The model presented here is a modified version of a standard sky model, which is the isotropic diffuse model derived by Liu and Jordan^11^. The isotropic diffuse model assumes that all diffuse irradiation is isotropic, meaning that the intensity of diffuse irradiation is uniform over the skydome. The irradiation on a tilted PV module composed of three components; beam, isotropic diffuse, and diffusely reflected from the ground. The beam irradiation is the direct irradiation that comes from the Sun without scattering by the atmosphere. The diffuse irradiation is the irradiation received from the Sun after scattering by the atmosphere, and it is harder to estimate since it depends on the cloudiness and the clearness of the atmosphere^7^. The last component of the isotropic model is the ground reflected irradiation by the surface of the earth and by any other surroundings. By combining three components as proposed by Liu and Jordan^9^, the solar irradiation on a tilted surface can be found as follows:

1 $$I_{t} = I_{b} R_{b} + I_{d } \left( {\frac{1 + cos\beta }{2}} \right) + I\rho_{g} \left( {\frac{1 - cos\beta }{2}} \right),$$

where $I_{b}$ is the hourly beam irradiation on a horizontal surface and $R_{b}$ is the ratio of hourly beam irradiation on the tilted surface to that on the horizontal surface^12^. $R_{b}$ is different for the rear surface, and in our methodology, it is determined by the symmetry of the path of the solar irradiation concerning the passage of time. The second component of the Eq. (1) refers to the diffuse irradiation on a tilted surface where $I_{d }$ is the hourly diffuse irradiation on a horizontal surface, $\beta$ is the tilt angle of the module, and $\left( {\frac{1 + cos\beta }{2}} \right)$ is the view factor from collector to the sky^13^. The last component is the ground reflected contribution where *I* is the global solar irradiation on the horizontal surface, $\rho_{g}$ is the ground reflectance (albedo) and the third multiplier is the view factor from collector to the ground.

### Model description

**Figure 3:**
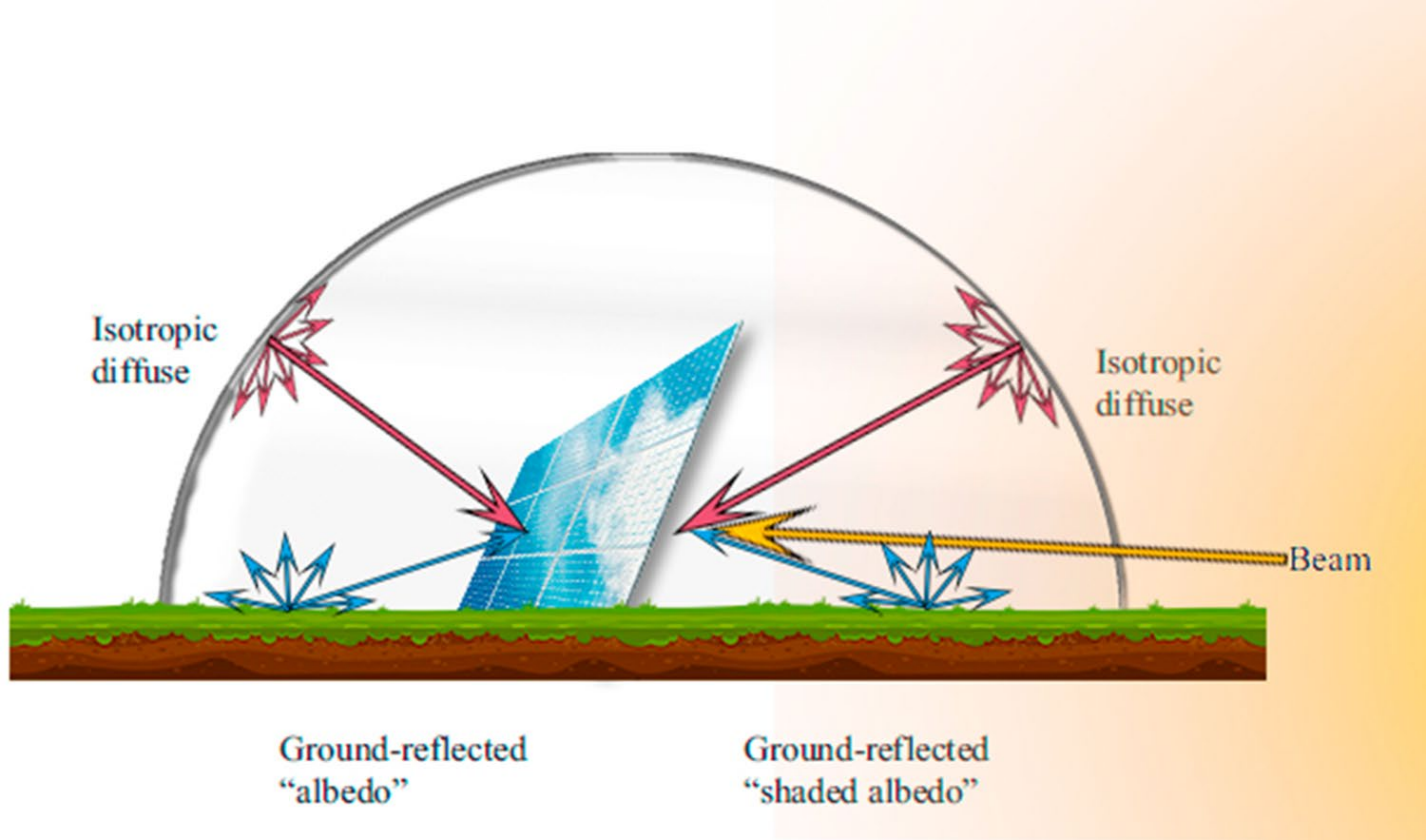
Components of solar irradiation incident on a bifacial PV module.

In this work, we have modified the Liu and Jordan's isotropic diffuse model for the estimation of the rear side irradiation. The first modification of the model is the tilt angle, instead of *β*, the complementary of *β* is taken as a tilt angle. The reason behind this modification is that the rear side is treated as a front side, so the tilt angle of the rear side is the complement of *β* (π–*β*). This correction contributes to the ground reflected irradiation component (the third component in Eq. 1) higher than the one in the original version as expected. Figure 3 represents the components of the solar irradiation incident on a bifacial PV module.

The second modification is altering the ratio of beam irradiation on the rear side of the bifacial PV module. The angle of incidence and the ratio of beam irradiation for the front surface have been calculated according to Ref.^9^, which includes parameters such as the latitude, declination, tilt angle, hour angle, and the surface azimuth angle. For the hours when the angle of incidence is higher than 90°, meaning that the Sun is on the rear surface, the following *R*_*b,front*_ values are taken as the ratio of beam irradiation on the rear surface *R*_*b,back*_. The beam contribution on the rear surface is due to the symmetry of the path of the Sun. There is only beam contribution on the rear surface for the sunrise and the sunset hours. An example set of the *R*_*b,back*_ calculation for an arbitrary day, is given below in Table 3. The first and last two numbers are for the hours that the Sun shines to the rear side. For these hours, the numbers in the last column are the numbers we use for *R*_*b,back*_, which are placed using the symmetry consideration above.

**Table 3:** *R*_*b,back*_ calculation between the sunrise and the sunset for an arbitrary day.

Hour	*R*_*b,front*_	*θ*	*R*_*b,back*_
05:00	0.00	113.34	0.66
06:00	0.00	100.31	0.21
07:00	0.21	86.89	0
08:00	0.66	73.3	0
09:00	0.83	59.69	0
10:00	0.92	46.27	0
11:00	0.97	33.45	0
12:00	0.99	22.43	0
13:00	1.00	17.25	0
14:00	0.99	22.43	0
15:00	0.97	33.45	0
16:00	0.92	46.27	0
17:00	0.83	59.69	0
18:00	0.66	73.30	0
19:00	0.21	86.89	0
20:00	6.66	100.31	0.21
21:00	3.96	113.34	0.66

In this study, all calculations are done for the hours between the sunrise and the sunset. Negative values of *R*_*b*_*,*_*front*_ have been taken as zero.

The last alteration comes from the relation between the ground reflected irradiation and the elevation of different parts of the rear side of the module^14^. Since different parts receive differing amounts of the ground reflected irradiation, a correction factor might be used for the rear illumination.

2 $$I_{t,back} = I_{b} R_{b,back} + I_{d } \left( {\frac{1 - cos\beta }{2}} \right) + I\rho f\left( h \right)\left( {\frac{1 + cos\beta }{2}} \right) .$$

To account for the differences between these irradiation values, a distribution function of elevation,$f\left( h \right),$ can be used from which an average value for the correction factor can be deduced. This function should be integrated in the interval of 0.5–1.16 m (min. and max. height of the module from the ground, respectively). One may use different distribution functions to find an average correction factor provided that the parameter of the function is determined using the system parameters in hand. We have used an exponential distribution function as the rear surface irradiation from bottom to up does not increase uniformly, and since the natural processes follow the natural logarithm.

3 $$f\left( h \right) = 1 - e^{{ - l/l_{c} }} .$$

Critical length constant *l*_c_ can be calculated by assuming that the value of the function at mid-length of 0.84 m to be 0.5:

4 $$0.5 = 1 - e^{{ - 0.84/l_{c} }} ,$$

which then gives:

$$l_{c} = 1.21.$$

To find an average value for this factor one can use the following averaging integration:

5 $$f\left( h \right) = \mathop \smallint \limits_{0.5}^{1.16} \left( {1 - e^{l/1.21} } \right)dl,$$

which gives:

$$F\left( l \right) = 0.33.$$

Here we assumed that the average correction factor is normalized out of the maximum possible correcting value of 1. Thus, for a bifacial solar module, we have found that average correction factor $f\left( h \right) = 0.33$. Using this value in Eq. (2), we reached the model calculation of solar irradiation impinging on the rear surface as:

6 $$I_{t,back} = I_{b} R_{b,back} + I_{d } \left( {\frac{1 - cos\beta }{2}} \right) + 0.33I\rho \left( {\frac{1 + cos\beta }{2}} \right) .$$

### Bifacial PV yield calculation

In order to make feasibility studies, it is crucial to estimate the yield of a PV module or an array. Many tools enable us to calculate the energy yield of a commercial (monofacial) PV module. In this study, we have used a modified version of PVForm^15,16^. Since there is not a commercially available extension tool for the bifacial PV module, we have used the same methodology for the rear surface and treat the rear surface the same with the front.

PVForm calculates the plane-of-array irradiance according to a modified version of the Perez 1990 algorithm^17^ and treats the diffuse radiation as isotropic for the zenith angles between 87.5° and 90°^18^.

7 $$I_{poa} = I_{b} + I_{d,sky} + I_{d,ground} .$$

However, there is an angle of incidence (AOI) correction that applies to incidence angles higher than 50°. The reason is to find the transmitted irradiance by considering reflection losses, and it is calculated as follows^18^:

8 $$f = b_{0} + b_{1} \theta + b_{2} \theta^{2} + b_{3} \theta^{3} + b_{4} \theta^{4} + b_{5} \theta^{5} ,$$

9 $$I_{tr} = I_{poa} - \left( {1 - f} \right) I_{b} {\cos}\left( \theta \right),$$

where $b_{0} , b_{1} ,b_{2} ,b_{3} ,b_{4} ,b_{5}$ are module cover polynomial coefficients and equal to 1.0, − 2.438E−3, 3.103E−4, 1.246E−5, 2.112E−7, − 1.359E−9 accordingly. $I_{tr}$ is the transmitted irradiance, $I_{b}$ is the beam component, and $\theta { }$ is the angle of incidence.

We have integrated a thermal model developed by Fuentes^19^ into the presented model to find the cell temperature. To calculate the operating cell temperature, the total plane-of-array irradiance, wind speed, and dry bulb (or ambient) temperature have been used according to the model. The model calculates the DC power output by adjusting the array efficiency for the irradiation values that are less than 125 W/m^2^.

10 $$P_{dc} = \frac{{I_{tr} }}{1000}P_{dc0} \left( {1 + \gamma \left( {T_{cell} - T_{ref} } \right)} \right) \quad I_{tr} > 125 \,{\text{W}}/{\text{m}}^{2} ,$$

11 $$P_{dc} = \frac{{0.008 I_{tr}^{2} }}{1000}P_{dc0} \left( {1 + \gamma \left( {T_{cell} - T_{ref} } \right)} \right) \quad I_{tr} \le 125 \,{\text{W}}/{\text{m}}^{2} .$$

The temperature coefficient $\gamma = - 0.5\%$, $T_{ref} = 25 \,^\circ {\text{C}}$ and $P_{dc0}$ is the nameplate DC rating.

There are a few modifications that we have done for the estimation of bifacial PV yield. First, the declination and the angle of incidence values are calculated by using more accurate Eqs. (2) and (4), respectively. Secondly, we have calculated $I_{poa}$ for the front and the rear surface (namely, $I_{t}$ and $I_{t,back}$ in our context) by using Eq. (1) and the derived Eq. (6) of the present work, and then, $I_{tr}$ and $I_{tr,back}$ is calculated. For $I_{tr,back}$ calculation, instead of filtering data for the incident angles, we have applied the AOI correction for the hours when there is a beam irradiation incident on the rear surface, meaning that $R_{b,back}$ is greater than zero. After finding the DC power output for both surfaces, we have added them up to find the total DC power output.

12 $$P_{dc,total} = P_{dc,front} + P_{dc,back} .$$

## Results and discussion

**Figure 4:**
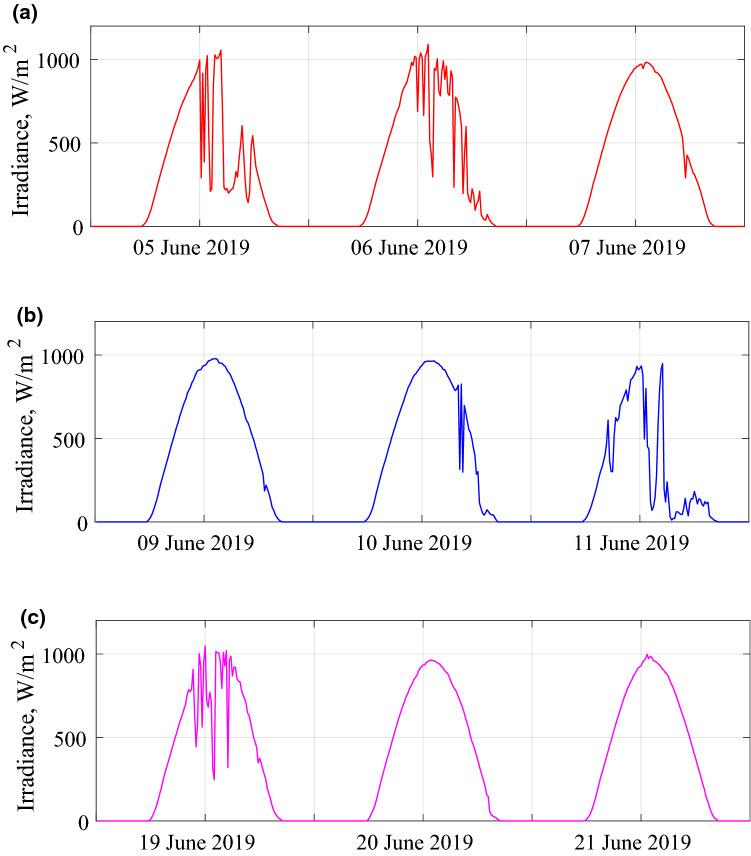
Horizontal irradiation measurements simultaneously with rear side irradiation measurements for (**a**) first configuration (**b**) second configuration (**c**) third configuration.

Horizontal irradiance is measured by Davis instrument, while the rear side irradiance is measured by using pyranometers as shown in Fig. 2. The days that we carried out these measurements are presented in Fig. 4a–c, which correspond to the configurations (a), (b), and (c) of Fig. 2, respectively.

The difference between the irradiation reaching the bottom, middle, and the top region of the rear side is also crucial in the modeling approach. That is, the analysis of this difference might be useful, yet this is our further research of interest.

**Figure 5:**
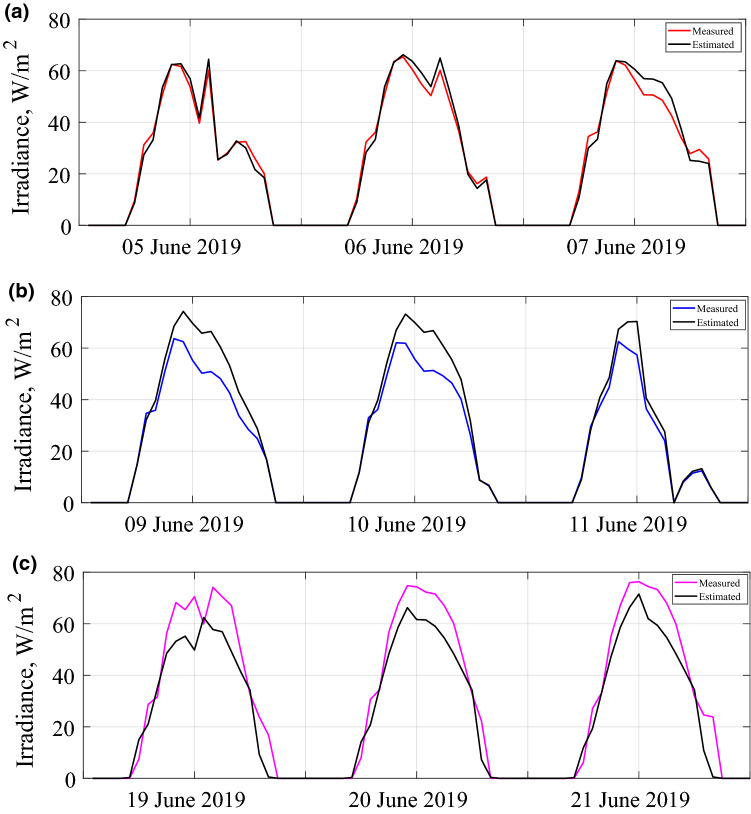
Measured and estimated rear side irradiations for (**a**) first configuration (**b**) second configuration (**c**) third configuration.

**Figure 6:**
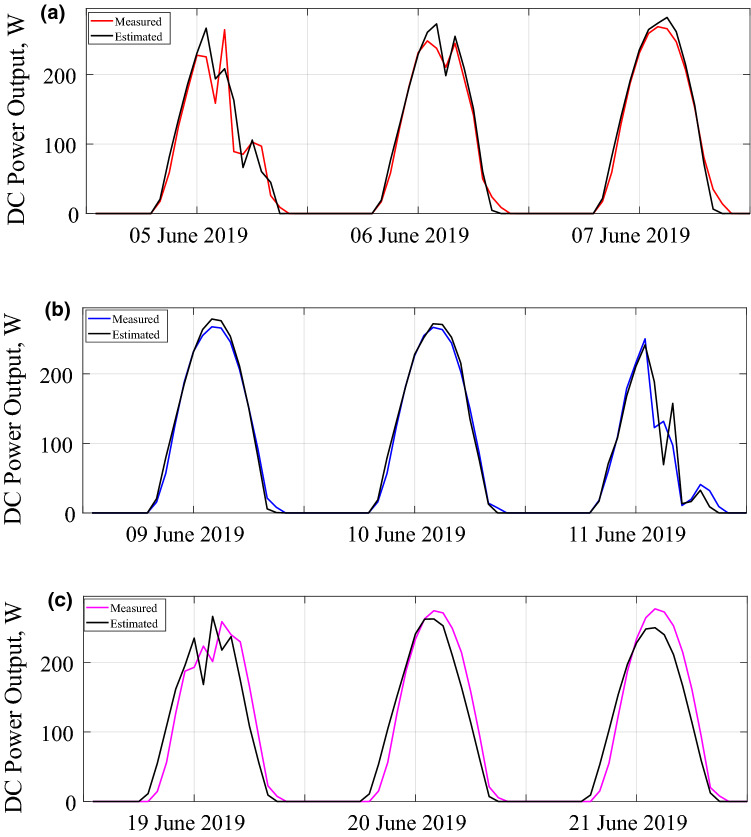
Measured and estimated DC power outputs for (**a**) first configuration (**b**) second configuration (**c**) third configuration.

The solar irradiation incident on the rear side of the bifacial PV module is calculated by modifying the Liu and Jordan's isotropic sky model, as explained above. Figure 5a–c give the measured average values of the rear side irradiation and the estimated values using the modeling approach described above for three days. The exhibited model estimates the DC power output of the tested module. Figure 6a–c show the measured and estimated values of the power output for three days.

By using two statistical methods such as Mean Bias Error (MBE) and the Root Mean Square Error (RMSE), the presented model is verified for three configurations. Besides, to make the verification of the model more comprehensive, the annual energy yield of the bifacial PV module is estimated.

The results showed that estimated and measured rear side solar irradiance values agree quite well. From statistical error calculations of the rear side irradiation estimations, the minimum and maximum values of MBE are − 0.08 W/m^2^ and − 5.53 W/m^2^, respectively. The minimum and maximum RMSE values for the same estimation are 1.95 W/m^2^ and 9.73 W/m^2^. There is a slight underestimation for the third configuration. The reason is that the lower and upper parts of the module receive more illumination than the middle section. As can be observed in Fig. 5b, when the pyranometers are placed at the middle and top rear side, the model slightly overestimates the data. The reason for the lower measured irradiance is the shading in the central part of the module. For a sunny day, the module itself and the nearby modules cast shadows in the middle. Therefore, the pyranometer measures the irradiance that is reflected from the shadow, not from the ground. The model does not consider the shading by the concrete ground. However, the overestimation is within acceptable levels. Nevertheless, the shading effect on the ground reflection is another research outcome of the present work about bifacial modules and arrays. That was already demonstrated in the Refs.^20^^,^^21^. For bifacial PV yield estimation, we have found that the minimum and maximum values of RMSE of the energy yield of a bifacial PV module are approximately 7.0 W/h and 30 W/h. For the MBE verification of the same estimation, the minimum value is 0.26 W/h, and the max value is − 5.6 W/h. The underestimation on the rear side irradiance for the third configuration leads to an underestimated power output as well. However, the estimates are much better for monthly and yearly estimation of the energy yield. For an annual estimation of the energy yield, the model has an approximately 1.4% error rate. We can state that the model outlined in the present study can be used to reach the efficiency and energy yield of bifacial modules and arrays.

## Conclusion

A model is presented for estimating the rear side irradiation of a single bifacial PV module. The measurements show that the top and bottom back of the module receives more sunlight than the middle part due to the shading. The model is based on the isotropic sky model of Liu and Jordan. For a single bifacial module installed at METU-GÜNAM, the model estimates are in good agreement with measured rear side irradiation not only for sunny days but also for cloudy days. Unlike the view factor calculations or the ray-tracing approach for the rear side irradiation estimation, the model presented here does not require high computational power. The model contains a correction factor calculated using a distribution function. The calculated value of the correction factor is 0.33. However, better value can be obtained by comparing the theoretical calculation using the long term such measurements at different climatic conditions. For annual bifacial PV yield estimations, the model has a relative percent error approximately equal to 1.4%. The presented model can be inserted into the efficiency calculations of bifacial PV modules/arrays or can be used for long term simulation purposes.
